# Fresh Waters and Fish Diversity: Distribution, Protection and Disturbance in Tropical Australia

**DOI:** 10.1371/journal.pone.0025846

**Published:** 2011-10-06

**Authors:** Stephanie R. Januchowski-Hartley, Richard G. Pearson, Robert Puschendorf, Thomas Rayner

**Affiliations:** 1 Australian Research Council Centre of Excellence for Coral Reef Studies, James Cook University, Townsville, Queensland, Australia; 2 School of Marine and Tropical Biology, James Cook University, Townsville, Queensland, Australia; 3 Australian Wetlands and Rivers Centre, School of Biological, Earth and Environmental Sciences, University of New South Wales, Sydney, New South Wales, Australia; Biodiversity Insitute of Ontario - University of Guelph, Canada

## Abstract

**Background:**

Given the globally poor protection of fresh waters for their intrinsic ecological values, assessments are needed to determine how well fresh waters and supported fish species are incidentally protected within existing terrestrial protected-area networks, and to identify their vulnerability to human-induced disturbances. To date, gaps in data have severely constrained any attempt to explore the representation of fresh waters in tropical regions.

**Methodology and Results:**

We determined the distribution of fresh waters and fish diversity in the Wet Tropics of Queensland, Australia. We then used distribution data of fresh waters, fish species, human-induced disturbances, and the terrestrial protected-area network to assess the effectiveness of terrestrial protected areas for fresh waters and fish species. We also identified human-induced disturbances likely to influence the effectiveness of freshwater protection and evaluated the vulnerability of fresh waters to these disturbances within and outside protected areas. The representation of fresh waters and fish species in the protected areas of the Wet Tropics is poor: 83% of stream types defined by order, 75% of wetland types, and 89% of fish species have less than 20% of their total Wet Tropics length, area or distribution completely within IUCN category II protected areas. Numerous disturbances affect fresh waters both within and outside of protected areas despite the high level of protection afforded to terrestrial areas in the Wet Tropics (>60% of the region). High-order streams and associated wetlands are influenced by the greatest number of human-induced disturbances and are also the least protected. Thirty-two percent of stream length upstream of protected areas has at least one human-induced disturbance present.

**Conclusions/Significance:**

We demonstrate the need for greater consideration of explicit protection and off-reserve management for fresh waters and supported biodiversity by showing that, even in a region where terrestrial protection is high, it does not adequately capture fresh waters.

## Introduction

Fresh waters are the most threatened ecosystems in the world, with high species extinction rates resulting from human dependence on freshwater resources, combined with localized and distant disturbances from upstream drainage networks, and further exacerbated by anthropogenic climate change [1]. The poor condition and vulnerability of freshwater ecosystems to human-induced disturbances is further amplified by the poor level of protection afforded to these ecosystems and the species they support (e.g. [2]–[5]). While protected areas act as a valuable tool in preventing habitat and biodiversity loss [6], and existing international commitments [7] are in place to establish protected area systems that contain viable representations of terrestrial, freshwater and marine ecosystems, freshwater protected areas remain rare [8].

There have been three reasons given in the freshwater conservation planning literature (e.g. [4], [9], [10]) for why these ecosystems have been poorly protected. Firstly, fresh waters are generally only protected incidentally through their incorporation into terrestrial protected areas [4], [10]. Secondly, partial inclusion of fresh waters within protected areas does not ensure protection as impacts outside protected area boundaries can have negative consequences [11]. Thirdly, the connectedness of freshwater ecosystems has offered unique challenges when it comes to planning and implementing protection [9]. In regions where there are no freshwater protected areas, these challenges can be addressed through systematic assessments that detail the effectiveness of terrestrial protected areas for representing freshwater ecosystems and biodiversity, and accounting for the limitations of partial inclusion and the connected nature of freshwater ecosystems.

Apart from three studies ([4] [12] [13]), previous assessments of freshwater ecosystem representation in terrestrial protected areas (e.g. [2], [5]) have focused solely on protection per se. However, given the interconnected nature of freshwater ecosystems and the limited explicit protection afforded to them, comprehensive evaluations need to take into account the disturbances that might affect them. The identification of disturbances and their proximity to protected areas can further demonstrate the level of effectiveness of terrestrial protected areas for abating threats to freshwater ecosystems and species [13].

This study presents a regional assessment of protection and human-induced disturbances to fresh waters and supported fish species in the Wet Tropics of Queensland, Australia, which is a notionally highly protected region. This research addresses a large knowledge gap ([14]–[17]) regarding the mismatch in basing management policies and conservation strategies for tropical streams on research in the temperate zone [e.g.18]. To date there have been few studies dealing with the systematic assessment of protection for fresh waters in Australia, with the majority of studies focused in temperate regions (e.g. [13], [19]–[21]). Apart from the work of Turak et al. (2011) [13] previous systematic assessments for fresh waters in Australia have either not been focused on protection (e.g. [22]) or have not given consideration to existing terrestrial protected areas [e.g.13, 19, 20]. Building on the existing network of terrestrial protected areas has been suggested as the most practical approach to improve freshwater ecosystem and species' representation in protected areas (e.g. [4], [23]). These assessments can be used further to guide the selection of additional protected areas to achieve both terrestrial and freshwater conservation objectives [4], [5], [10], [23].

We expand on previous assessments of terrestrial protected area effectiveness (e.g. [2], [3], [5]) and disturbances influencing the condition of fresh waters (e.g. [4]) by: 1) including tributaries as well as main river systems in our analysis; 2) accounting for the representation of fresh waters and supported fish species (not only rare species) in the terrestrial protected area network; 3) assessing the total amount of protection as well as the percent representation of stream order length, wetland area and the distribution of 45 fish species, protected entirely within IUCN category II protected areas (chosen as these protected areas are categorized with the highest level of formal protection to terrestrial ecosystems in the region, and by definition afford a high level of protection to ecosystem processes important for species persistence); and 4) quantifying current adjacent and upstream human-induced disturbances that influence condition of stream reaches and wetlands both within and outside of terrestrial protected areas. We focused on fish species because their taxonomy is well known, they are strongly dependent on stream and wetland ecosystems and because there was sufficient available data to model their current distributions. Protection level of each ecosystem and species was determined for the protected area categories for the State of Queensland and the IUCN, making our results both nationally and internationally relevant. Our results are an initial step towards identifying systematic conservation priorities for fresh waters and the biodiversity they support, at a regional scale.

## Results

### Stream reaches and wetlands

**Figure 1:**
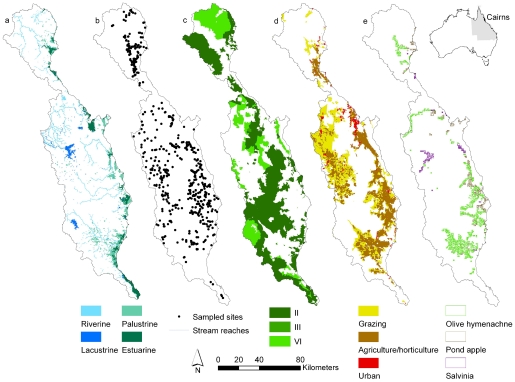
Distribution of wetlands, fish occurrences, protected areas, land use and invasive species. The Wet Tropics study area in north Queensland, Australia, showing the spatial distribution of: a) wetland types; b) stream reaches and sites sampled for fish; c) IUCN protected area categories; d) land uses and e) aquatic invasive species.

The stream network derived from the 30 m×30 m digital elevation model resulted in six stream orders (Figure S1). Palustrine and estuarine wetlands are distributed within the floodplains and coastal areas, while the lacustrine wetlands are distributed in the uplands (Figure 1a).

**Figure 2:**
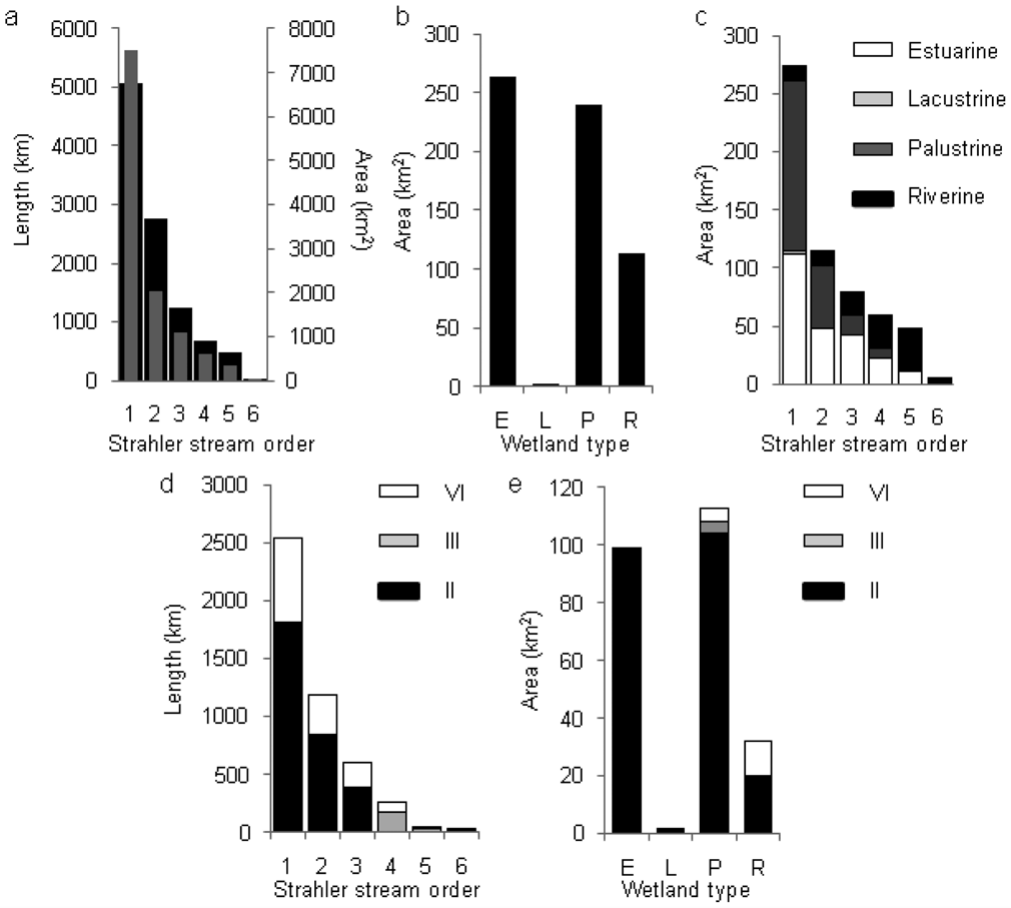
Statistics for stream reaches, sub-catchments and wetlands in the Wet Tropics. The total: a) length (grey bars) and area (black bars) of each Strahler stream order and adjacent sub-catchment; b) area of each wetland type; c) total area of the four wetland types in adjacent sub-catchments for each Strahler stream order; d) length of each Strahler stream order, with IUCN categories indicated; and e) area of each wetland type represented within IUCN categories II, III and VI.

Sub-catchments adjacent to streams in order 1 occupy the greatest area (7498 km^2^), while those adjacent to stream order 6 occupy the least area (40 km^2^) (Figure 2a). Estuarine wetlands occupy the greatest total wetland area (263 km^2^); lacustrine wetlands, the least (2 km^2^) (Figure 2b). Sub-catchments adjacent to order 1 streams support the greatest area of estuarine, lacustrine and palustrine wetlands (Figure 2c). The greatest area of riverine wetlands occurs in sub-catchments adjacent to stream order 5 (35 km^2^) and 4 (28 km^2^).

### Freshwater fish diversity

The average AUC (the area under the receiver operating characteristic (ROC) curve) value across all fish species predictions was 0.84. More than 85% of the 45 fish species had AUC >0.75 (Table S1), indicating that the MARS model had strong discriminatory power. The maximum number of fish species predicted to occur in any one stream reach was 21; the minimum was 2. Areas of high fish species richness occur in stream orders 5 and 6 on the coastal plains (Figure S1).

### Stream reach and wetland protection

The greatest stream reach length and wetland area is protected under IUCN category II, National Parks (Table 1). Streams in order 1 have the greatest length protected (2537 km = 25%), while streams in order 6 have less than 1% of the total reach length protected (Figure 2d). All four wetland types have the greatest area within IUCN category II (223 km^2^ = 36%) (Figure 2e). Palustrine wetlands have the greatest area within IUCN category II protected areas (104 km^2^ = 91%). Less than 1 km^2^ of estuarine and lacustrine wetlands is protected in categories III or VI, and less than 1 km^2^ of riverine wetlands is within IUCN category III (Figure 2e).

**Table 1:** IUCN and State of Queensland protected areas.

IUCN category	Queensland protected area	Length	Area
II	National Park	3250	223
III	National Park	19	4
VI	Forest Reserve	366	4
	State Forest	440	7
	Timber Reserve	562	6

The total stream reach length (km) and wetland area (km^2^) protected in IUCN protected area management categories (IUCN category) and the State of Queensland's protected area classification in the Wet Tropics.

**Figure 3:**
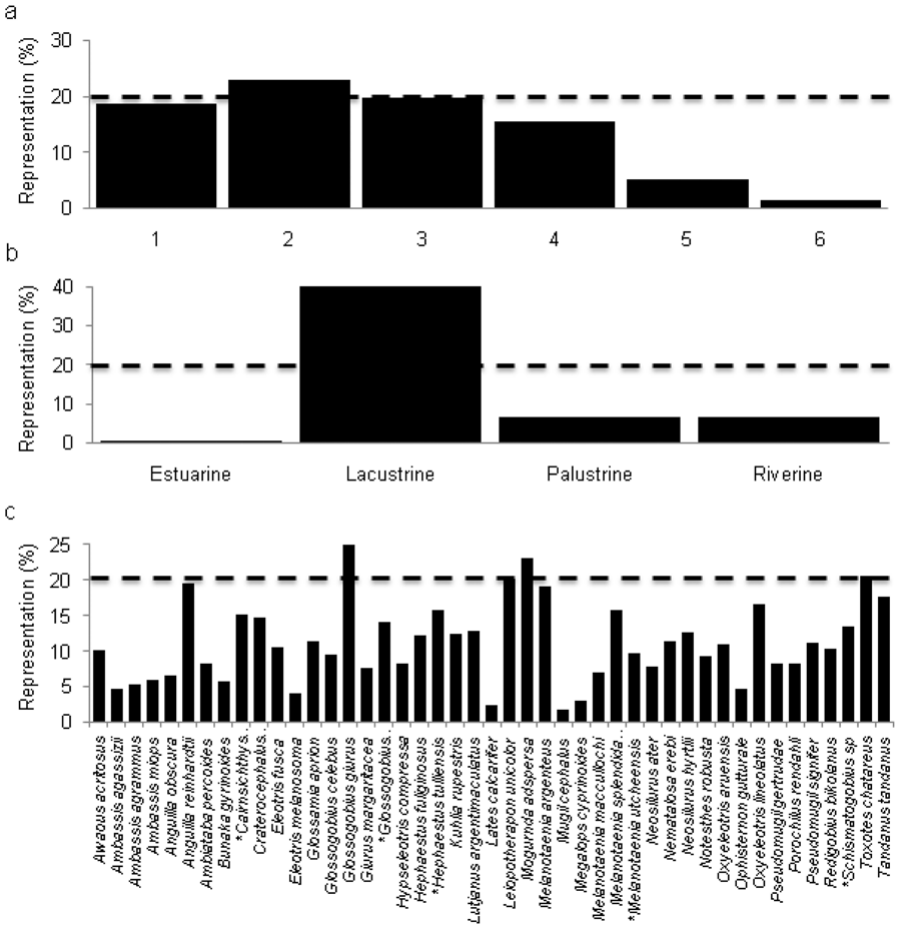
Representation of Strahler stream order sub-catchments, wetlands and fish in IUCN category II protected areas. The percent representation of: a) sub-catchment area adjacent to streams in Strahler stream orders 1–6; b) wetland types; and c) each fish species distribution occurring completely within an IUCN category II protected area. *  =  species that are endemic to the Wet Tropics. The dashed lines indicate 20% representation.

Only streams in order 2 achieved the minimum target of 20% representation fully within the IUCN category II protected areas (23%) (Figure 3a). Approximately two percent of sub-catchments adjacent to streams in order 6 are fully within an IUCN category II protected area (Figure 3a). Only lacustrine wetlands have greater than 20% of the total wetland area fully within an IUCN category II protected area (Figure 3b). Five of the 45 modeled fish species have at least 20% of their distribution represented in IUCN category II protected areas (Figure 3a). Twenty species have less than 10% of their Wet Tropics distribution represented, while the remaining 20 have between 10 and 19% of their distribution represented in IUCN category II protected areas. None of the endemic fish species included in our analysis have better than 15% of their Wet Tropics distribution in IUCN category II protected areas (Figure 3a).

### Land use and human-induced disturbances to fresh waters and biodiversity

**Figure 4:**
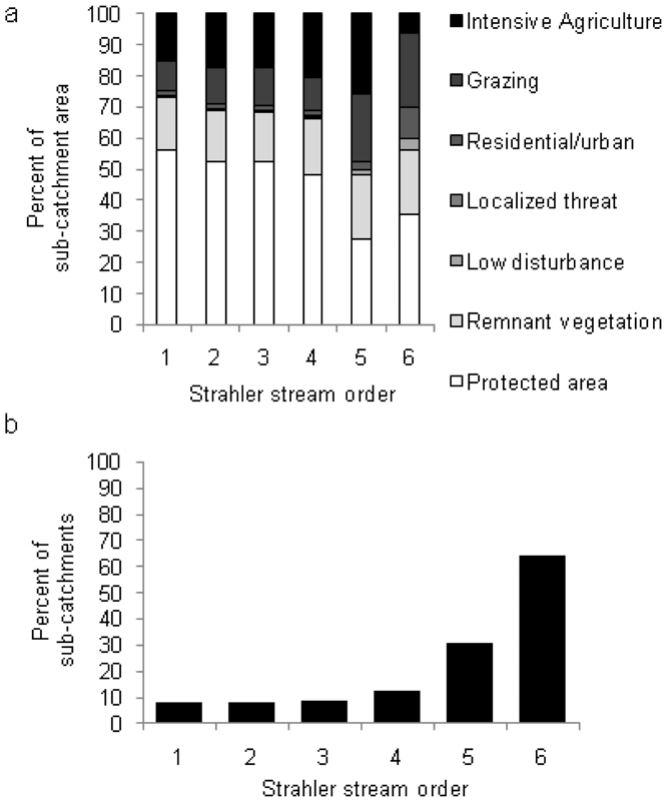
Human-induced disturbances: land use and invasive species. The percent of sub-catchment area adjacent to each Strahler streamorder 1–6 that is: a) covered with each of the seven land uses or b) covered with an invasive macrophyte: olive hymenachne (*Hymenachne amplexicaulis*), pond apple (*Annona glabra*) and/or salvinia (*Salvinia molesta*).

More than 50% of sub-catchments adjacent to streams in orders 1–3 are protected. Adjacent sub-catchments of order 5 stream reaches have the lowest percent area (27%) protected (Figure 4a). Sub-catchments adjacent to streams in order 5 have the highest percent area (48%) that is grazing or intensive agriculture or horticulture, while sub-catchments adjacent to streams in order 6 have the highest percent area that is urban/residential (10%) (Figure 4a). The highest percent of adjacent sub-catchments with weed infestations (olive hymenachne, pond apple, or salvinia) are of stream reaches in order 6 (Figure 4b).

**Figure 5:**
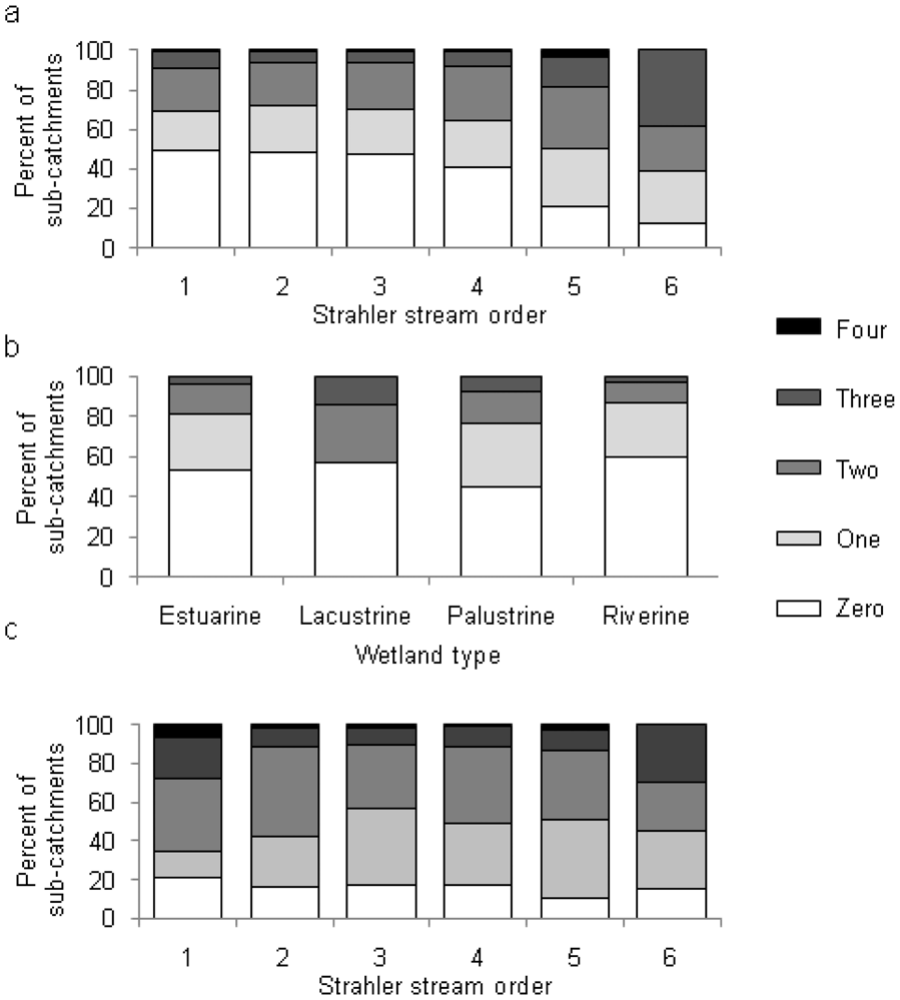
Multiple human-induced disturbances. The percent of: a) sub-catchments adjacent to Strahler stream orders 1-6; b) sub-catchments supporting each of the four wetland types; and c) sub-catchments adjacent to each Strahler stream order 1–6 that support at least 10 fish species, that have 0, 1, 2, 3 or 4 human-induced disturbances present.

There is a maximum of four human-induced disturbances found in any single sub-catchment. Stream order 5 has the highest percent (4%) of sub-catchments with four human-induced disturbances. Stream order 6 has the highest percent (38%) of adjacent sub-catchments with three human-induced disturbances (Figure 5a). Only sub-catchments supporting palustrine wetlands have four human-induced disturbances; these sub-catchments also have the highest percent (46%) occupied by one or more disturbances (Figure 5b).

Twelve percent of all stream reaches were modeled as having at least 10 fish species present. Sub-catchments adjacent to streams in order 1 that have at least 10 fish species present also have the highest percent of sub-catchments with no human-induced disturbances (Figure 5c). Nevertheless, those sub-catchments also have the highest percent with four human-induced disturbances (5%). Stream reaches in order 5 that support at least 10 fish species have the highest percent (90%) of sub-catchments occupied by one or more human-induced disturbance.

**Figure 6:**
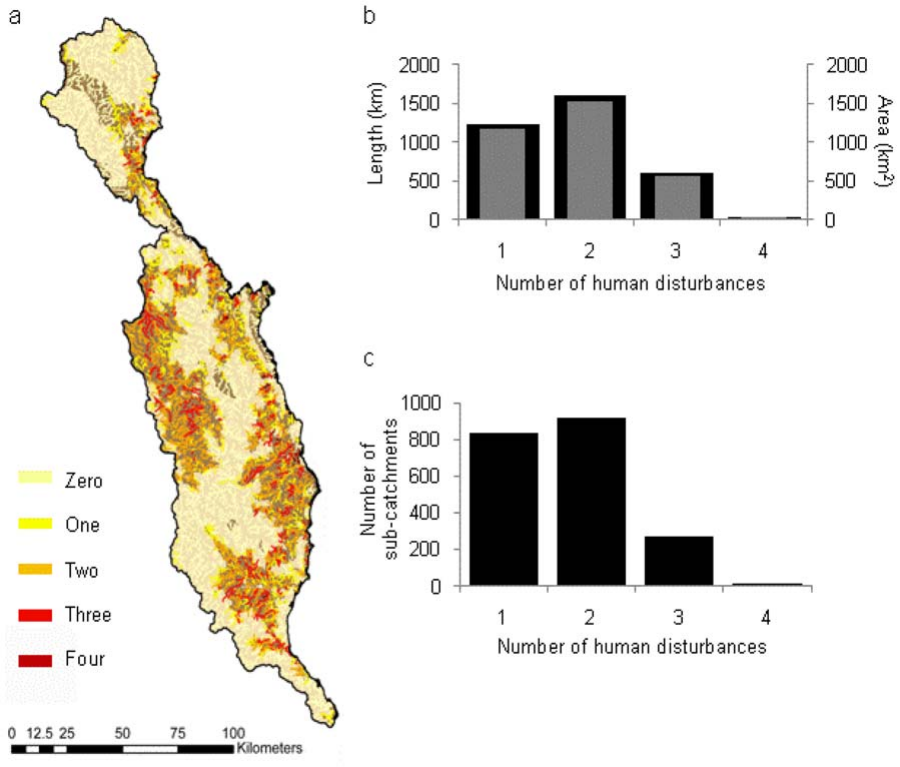
Multiple human-induced disturbances upstream of protected areas. The distribution and prevalence of human-induced disturbances upstream of protected areas by: a) the number of stream reaches with 0, 1, 2, 3 or 4 human-induced disturbances; b) the total stream length (grey bars) and sub-catchment area (black bars) with one or more human-induced disturbance; and c) the number of sub-catchments with one or more human-induced disturbance.

Sub-catchments upstream of protected areas have a variable number of human-induced disturbances present (Figure 6a). The greatest stream reach length (1518 km), sub-catchment area (1600 km^2^) and number of sub-catchments (919) upstream of a protected area have two human-induced disturbances present, while the least length (26 km), area (28 km^2^) or number of sub-catchments (11) have four disturbances present (Figure 6b,c).

## Discussion

Our results demonstrate that: 1) terrestrial protected areas do not afford effective protection to fresh waters and fish species; 2) higher-order stream reaches and their associated wetlands are influenced by the greatest number of human-induced disturbances and are also the least protected; and 3) terrestrial protected areas are subjected to a variable number of human-induced disturbances from upstream sub-catchments. Our results reflect global trends of the state of fresh waters and their supported biodiversity (e.g. [1], [24]), despite the high proportion of protected land area in the Wet Tropics. The poor protection of fresh waters in the Wet Tropics warrants attention with regard to policy, biodiversity planning and implementation of conservation actions. Planning for the conservation of fresh waters and their dependent species requires whole-of-catchment (or sub-catchment) consideration of connectivity and disturbances and simple place-based protection is inadequate. Our approach is a first step for identifying streams and wetlands that lie entirely within a protected area and that may act as a starting point for further protection or restoration.

Globally, there has been very little emphasis on proclaiming protected areas for the primary purpose of conserving fresh waters [4], [10]. It is therefore not surprising that our results demonstrate the inadequate representation of sub-catchments, wetlands and fish species. As in temperate regions of Australia [13] the majority of protection afforded to stream reaches and wetlands in the Wet Tropics is restricted to upland, mountainous areas. For example, lacustrine wetlands are the only wetland type with greater than 20% of its total area in the Wet Tropics represented completely in IUCN category II protected areas, but are likely to have been protected by default given their iconic nature (they comprise mainly isolated crater lakes).

Although portions of streams and wetlands are protected within the current protected area network, fragments of stream reaches, sub-catchments or wetlands do not constitute a comprehensive protected area network for fresh waters [4]. Furthermore, inclusion in protected areas does not guarantee conservation. While almost all of the palustrine wetlands listed under IUCN category II protection in the Wet Tropics are listed as endangered, their protection is highly fragmented. Only 7% of the total area of these wetlands lies completely within an IUCN category II protected area. In addition, palustrine wetlands lying in the floodplains of most catchments of the Wet Tropics have historically been filled or have had riparian vegetation heavily cleared for agriculture (e.g., sugar cane). Many of the palustrine wetlands are or were endemic to the region, and, those remaining in the landscape are highly endangered, and as we have demonstrated, susceptible to landscape alterations and weed infestations [25]. Specifically, palustrine and estuarine wetlands are of particular interest to the international conservation community as they are important for freshwater biodiversity and ecosystem services, and are the types of aquatic ecosystems highlighted as priorities for protection as a result of the Convention on Biodiversity 10th Conference of the Parties in 2010 [7]. Given that IUCN category II protected areas afford the greatest level and area of protection to wetlands in the Wet Tropics there is clearly a need for greater conservation action to protect, restore and maintain ecosystem functioning of these wetlands in the region. This would not only meet international conservation targets, but also ensure conservation of critical habitats that support a number of endemic and range-restricted species in the Wet Tropics itself.

Fish species are poorly represented in the Wet Tropics protected area network as has been noted in previous assessments of protection gaps for freshwater fish [2], [5]. Protected areas primarily occur in areas of higher elevation, while most freshwater fish species occur only in the lowlands. None of the endemic fish species we modeled have 20% of their Wet Tropics distribution within an IUCN category II protected areas. This is a major concern not only for the endemic and rare species that we were able to model, but especially for those species that we could not model, because of their rarity or restricted distribution in the Wet Tropics. The rarity and endemicity of many fish species in the Wet Tropics may warrant greater conservation action than at present. Many fish species could be at high risk because their prime habitat is in the poorly protected floodplain and coastal waterways.

Our results concur with others [4], [13] in demonstrating that the six human-induced disturbances we evaluated increase the vulnerability of fresh waters and fish species both within and outside of protected areas in the Wet Tropics. We demonstrate that fresh waters within protected areas of the Wet Tropics are especially vulnerable to exogenous disturbances. For example, our results show that 3 288 km (32%) of stream reach length upstream of protected areas have at least one human-induced disturbance present. The continuous nature of fresh waters makes them particularly susceptible to external disturbances even if portions of a stream reach or wetland are protected [4], [13], [20]. Therefore, while some stream orders have more protection than others, the distribution of protection does not necessarily reduce threats to these systems, or to the species they support.

### Management implications

The inadequacy of the Wet Tropics protected-area network in representing important freshwater ecosystems and species underscores the need for freshwater-specific conservation. The terrestrial protected area includes a large proportion of the Wet Tropics (approximately 60%), yet its spatial distribution is far from optimal in providing adequate coverage of fresh waters and the fish species they support, especially endemics.

A major challenge to quantifying the effectiveness of protected areas for representing species is the lack of data available. Not unlike other tropical regions that support most of the world's species [15], [24], the information on freshwater biodiversity is incomplete in the Wet Tropics. We were able to account for the effectiveness of terrestrial protected areas for representing many freshwater fish species as data were available; however, data are inadequate for other taxa, such as many invertebrate groups, frogs, reptiles and birds that are also reliant on fresh waters but whose distributions across catchments differ markedly from those of most fishes. Thus it is likely that the protected area network is effective at protecting species of invertebrates that are reliant on the headwater streams, which are better protected than most of the other stream orders. Given the differences in distribution and habitat dependence of different taxa, regional assessments of the effectiveness of protected areas for the full complement of taxa would be beneficial as it is unlikely that any one taxon can act as a surrogate for the whole biota.

Although the existing protected area network in the Wet Tropics does not include broad representation of fresh waters, the current network can provide starting points to establish further protection or to link existing undisturbed areas with other critical areas through restoration. Protection of an entire catchment may be preferred from a conservation standpoint, but this is rarely feasible given the multiple demands on resources that catchments and fresh waters experience [10], [26]. Consequently, there is a need for off-reserve management of fresh waters on both public and private lands. Given that resources for management are typically limited, an important first step is to identify fresh waters and species that are particularly vulnerable to local and upstream/downstream disturbances [4], [20].

Following the terminology and approaches proposed by Abell et al. (2007) [10] we suggest the need for a combination of place-based and whole-of-catchment management strategies to ensure functional aquatic ecosystems in the Wet Tropics and comparable regions. Firstly, systematic approaches [6] would be used to identify place-based focal areas that complement existing protected areas, but that could be set aside for specific freshwater ecosystems or species that require protection. Secondly, we suggest identifying critical management zones that complement and assist in maintaining functionality of identified focal areas, such as riparian zones where restoration of riparian vegetation and control of invasive species are being undertaken. Finally, we suggest the need to adopt catchment management zones for entire catchments upstream of critical management areas. Catchment management zones would also be a positive alternative to ‘locking up’ additional areas in formal protection, and would allow for productive lands to be utilized under best-management principles allowing for multiple uses and maintenance of ecosystem services.

There is a pressing need to consider the threat of global changes that are hard to plan for or manage. For example, in the Wet Tropics, rising sea levels are likely to reduce the extent of higher-order streams, which support the greatest diversity of fish. Changes in rainfall and cloud interception are likely to lead to increased variability of discharge, and reduced dry-season discharge, particularly in upland streams [27], and resultant changes in habitat are likely to negatively affect many endemic species, particularly riffle specialists among the invertebrates [28], fish [29] and frogs [30]. Holistic approaches to conservation that consider both place-based protection and whole-of-catchment management would provide a better buffer than place-based protection alone, encouraging ecosystem and species persistence under current pressures and anticipated global change.

Given the high proportion of the Wet Tropics landscape that is protected, it might be expected that the protection of fresh waters in this region would be much higher than in other regions, especially in the tropics. However, we have demonstrated both the limitation of terrestrial protected areas for effectively protecting fresh waters and their supported biodiversity, and the failure of these protected areas in abating threats to these systems. Existing freshwater protected areas (e.g., Ramsar wetlands) often do not afford effective protection as protected areas lie downstream of disturbances [10], and little consideration is given to upstream protection or management to mitigate disturbance. Moreover, wetland protection tends to focus on specific sites (especially lentic systems) and ignores the interconnected network across catchments. We have demonstrated that protected areas cannot act as the only strategy for achieving freshwater conservation challenges. There is a need to build on existing protected areas networks to provide protection to focal freshwater ecosystems, and connect this with whole-of-catchment management.

## Materials and Methods

### Study Area

The Wet Tropics bioregion comprises a narrow strip of land (<80 km wide) on the north-eastern coast of Queensland, Australia (Figure 1a–e) and is defined by its climate and vegetation. The climate is characterized by a monsoon-dominated wet season [31], with reliable rainfall through the rest of the year, resulting in the highest average annual rainfall in Australia (>8000 mm/year on mountain tops). Landforms include a mountain range that runs parallel with the coast and roughly perpendicular to prevailing south-east trade winds, a fertile plateau (tableland) within the mountain range, and a narrow coastal plain. The vegetation of the mountains is mainly rainforest, while vegetation of the coastal plain, originally comprising open forests, rainforest and wetlands, is now mostly cleared for grazing, agriculture and horticulture [31]. There are nine main rivers exclusively within the Wet Tropics: from north to south they are the Daintree, Mossman, Barron, Mulgrave, Russell, North Johnstone, South Johnstone, Tully and Murray Rivers. A tenth Wet Tropics river, the Herbert, has much of its catchment outside this bioregion, and was excluded from our analyses. The nine catchments drain a total area of 11 862 km^2^ into the Great Barrier Reef lagoon.

One of the major impacts to fresh waters in this region has been the degradation and loss of riparian forests from wetland and floodplain habitats [29], commonly accompanied by invasion by introduced plant species [29]. Beyond these local and regional impacts on freshwater ecosystems biodiversity there are several climate-related global impacts such as rising sea levels, reduced rainfall, and reductions in mountain rainforest cloud interception (e.g. [27]) that are predicted to influence freshwater habitats, stream flows, species diversity, and endemicity.

### Stream reaches and wetlands

We compiled available spatial data for fresh waters in the Wet Tropics, including: stream reaches and wetlands (Figure 1a), adjacent sub-catchments, and upstream catchment areas for each stream reach. We derived stream reaches (n = 7210) from a 30 m×30 m digital elevation model (approximately 1: 100 000 scale mapping) [32] using ArcHydro 1.1 [33] in ArcGIS® 9.3 (Environmental Systems Research Institute 2009). We assigned a Strahler stream order to each mapped stream reach using ArcGIS 9.3. We used Strahler stream order as a surrogate for representing stream size, which is arguably one of the most fundamental determinants of stream ecosystem structure and function [34], [35].

For each of the mapped stream reaches (Figure 1b) we also determined the adjacent sub-catchment (defined as the area that drains into the stream reach, located directly next to the reach, not upstream) and upstream catchment area (defined as the upstream area draining each stream reach, apart from first order streams as the upstream area does not differ from the adjacent sub-catchment area) using ArcHydro 1.1 in ArcGIS 9.3. The wetland types were defined and mapped at a scale of 1: 50 000 by the Queensland Department of Environment and Resource Management (DERM) [25]. For subsequent analyses we used four broad wetland types (Table 2) as well as sub-catchments where the wetlands occur to summarize adjacent disturbance pressures. We chose this broad classification of wetlands to allow for comparison of our results in other regions in Australia and the tropics. Using ArcGIS 9.3, we determined: 1) the total stream reach length (km) and adjacent sub-catchment area (km^2^) for each stream order; 2) the total area (km^2^) of the four wetland types; and 3) the total number of occurrences for each of the four wetland types in sub-catchments of each stream order.

**Table 2:** Definitions of wetland types and conservation status.

Wetland Type	Definition
Riverine	Riverine wetland or fringing riverine wetland. These are wetlands with an open, non-vegetated channel.
Lacustrine	Lacustrine (lakes). These are generally larger than 8 ha, situated in a topographic depression or dammed river channel and have <30% vegetation cover.
Palustrine	Palustrine (swamps, marshes etc). These are generally non-tidal areas dominated by vegetation (>30% cover) or, if lacking vegetation, area <8 ha.
Estuarine	Estuarine wetlands. Intertidal areas such as mangroves and salt flats.

Conservation Status	Definition
Endangered	The area of remnant vegetation for the regional ecosystem is <10% of the pre-clearing extent of the regional ecosystem; or the area of remnant vegetation for the regional ecosystem is 10–30% of the pre-clearing extent of the regional ecosystem and <10 000 ha.
Of concern	The area of remnant vegetation for the regional ecosystem is 10–30% of the pre-clearing extent of the regional ecosystem; or the area of remnant vegetation for the regional ecosystem is >30% of the pre-clearing extent of the regional ecosystem and <10 000 ha.
Least concern	The area of remnant vegetation for the regional ecosystem is >30% of the pre-clearing extent of the regional ecosystem and >10 000 ha.

Wetland definitions and conservation status from Queensland Department of Environmental Resource Management.

### Freshwater fish diversity

We used the Northern Australia Freshwater Fish Atlas database (http://www.jcu.edu.au/vhosts/actfr/Projects/FishAtlas/Index.htm),which is based on fish species presence/absence data collected between 1990 and 2009. Sampled stream reaches were well distributed across the Wet Tropics and representative of major catchments, instream habitats (runs, riffles, and pools), and length and width of reaches. From this database we selected species with strong association with fresh waters, including species also found in estuarine and marine systems. We eliminated duplicate records from sub-catchments to model only geographically unique occurrences. We also eliminated records older than 15 years and species with fewer than ten occurrences in the database to ensure adequate prevalence for modeling. The cleaned database contained records for 45 species from 448 of the7210 stream reaches in the nine selected catchments.

We modeled current distributions of the 45 fish species using 17 predictor variables that were available for all 7210 stream reaches (Table 3), including nine physical variables, four land-use variables and presence/absence of three invasive aquatic plants. The variables were attributed to stream reaches, adjacent sub-catchments, or the upstream catchment area flowing into stream reaches using ArcGIS 9.3 and ArcHydro 1.1. We considered these models to be representative of the 45 species' current distributions as we accounted for potential responses to disturbance as well as to natural gradients [36]). Therefore, species should only be predicted with high probabilities of occurrence in stream reaches that were in good condition or where the disturbances included in the models did not exceed the species' tolerance levels [36].

**Table 3:** Environmental variables and human-induced disturbances and respective attributed freshwater feature.

Environmental variable	Attributed feature
Stream length (km)	Stream reach
Stream order	Stream reach
Minimum elevation (m)	Stream reach
Maximum elevation (m)	Stream reach
Minimum slope (degrees)	Stream reach
Maximum slope (degrees)	Stream reach
Alluvium cover	Adjacent sub-catchment
Annual rainfall average (mm)	Adjacent sub-catchment
	Upstream catchment
Woody foliage cover	Adjacent sub-catchment
**Land use and invasive species**	
Remnant vegetation cover (km^2^)	Adjacent sub-catchment
Urban/residential cover (km^2^)	Adjacent sub-catchment
Grazing cover (km^2^)	Adjacent sub-catchment
Intensive agriculture/horticulture cover (km^2^)	Adjacent sub-catchment
Olive hymenachne (*Hymenachne amplexicaulis*) presence	Adjacent sub-catchment
Pond apple (*Annona glabra*) presence	Adjacent sub-catchment
Salvinia (*Salvinia molesta)* presence	Adjacent sub-catchment

Environmental variables and human-induced disturbances as well as their attributed features used for fish species distribution modeling.

We determined modeled fish distributions using MARS (multivariate adaptive regression splines). We built a single multi-response MARS model for all 45 species. The model was fitted using code provided by Elith and Leathwick (2007) [37] for the mixture and flexible discriminant analysis (MDA) library in the R statistical software package, Version 2.10.1 (R Development Core Team 2009). MARS is a method for non-parametric regression modeling, useful for addressing complex non-linear relationships between response and explanatory variables. MARS enables exploration of interactions between predictors and can fit a multi-response model which simultaneously relates variation in the occurrence of all species to the environmental predictors [38]. Multi-response species models have been shown to best recover overall variation in species composition compared to single-species models [39], because species that have been better sampled and represented in the dataset can help inform poorly sampled species [37], [40]. Several researchers have demonstrated the utility of multi-response MARS models for freshwater conservation planning (e.g. [40], [41]).

To validate the predictive model we used the area under the receiver operating characteristic (ROC) curve (AUC) [42]. The ROC addresses false-negative and false-positive predictions, and is quantified by the AUC. An AUC score of 0.5 indicates a model with no discriminatory ability while a score of 1 indicates that presences and absences are perfectly discriminated. A score of 0.60 or greater is generally considered an acceptable threshold for model performance [42]. We used a k-fold cross-validation procedure [42] to determine the AUC. The cross-validation divided the presence-absence data into 10 random subsets, successively removing a single data point from each subset and refitting the model with the remaining data, before predicting the omitted data and calculating the average AUC across all subsets.

### Protected area network

To determine the effectiveness of terrestrial protected areas for representing fresh waters and fish species we used spatial data on the Wet Tropics World Heritage Area and the protected areas of Queensland Estate provided by the Queensland Department of Environment and Resource Management [43] (Figure 1c). The Queensland government has defined four types of protected areas in the Wet Tropics: National Park, State Forest, Timber Reserve and Forest Reserve, each of which has a separate IUCN protected area management category [44]. IUCN protected area management categories were developed to provide a basis for international comparison and are assigned according to the primary management objective in the legal definition of each protected area. In the Wet Tropics the IUCN management categories present are: category II, which includes areas managed primarily for ecosystem protection and recreation; category III, which includes areas managed primarily for conservation of specific natural features (both categories II and III are National Parks in the Wet Tropics); and category VI, which is managed primarily for the sustainable use of natural ecosystems (e.g., State Forest, Timber Reserves and Forest Reserves). We used the Queensland Government's protected area listing and the IUCN management categories for subsequent analyses, allowing us to provide informative results for both regional and national decision makers, as well as a means for international comparisons on levels of fresh water protection.

### Land use and human-induced disturbances

We represented extant human-induced disturbances using spatial data on land use in 1999, provided by DERM [45] and aquatic invasive plants provided by Far North Queensland Regional Organization of Councils [41]. All land-use data were mapped at nominal scales of 1∶50 000 and 1∶100 000 and aquatic invasive plants were mapped in grids at a scale of 1 km×1 km. While there have been some changes in land use in the Wet Tropics Region since 1999, they have not been substantial.

We designated seven land-use categories: 1) protected areas (as defined above), 2) remnant native vegetation that is not protected, 3) low disturbance uses (energy, power lines), 4) localized disturbances (e.g. mining or wastewater treatment) which occupy <1% of the landscape, 5) urban/residential, 6) grazing, and 7) intensive agriculture or horticulture. We excluded waterways as a land use for our assessment, so all area-based calculations of land use were based on a total area of 11 618 km^2^. For subsequent analyses we considered urban/residential, grazing and intensive agriculture or horticulture land uses as human-induced disturbances as these are large-scale land uses that dominate the Wet Tropics landscape (Figure 1d) and are of particular interest when it comes to managing non-point source human-induced disturbance entering fresh waters as well as the downstream Great Barrier Reef Lagoon. Due to the small proportion of sub-catchments that are occupied by the low and localized disturbances we omitted them from subsequent analyses. This was not to disregard the potential local scale affects from low and localized disturbances. Rather, we wanted to focus on describing those large-scale human-induced land uses that are of particular concern to catchment, as well as land and marine managers in the region [46], [47].

Spatial data were available for three invasive macrophyte species that are known to have severe impacts on freshwater ecosystems across northern Australia: olive hymenachne (*Hymenachne amplexicaulis*), pond apple (*Annona glabra*) and salvinia (*Salvinia molesta*). We used existing mapped 1 km×1 km grids of olive hymenachne, pond apple and salvinia presence based on existing data and information from an expert workshop held by Far North Queensland Regional Organization of Councils [48]. We then attributed presence/absence of olive hymenachne, pond apple and salvinia to each of our adjacent sub-catchments for subsequent analyses.

### Protection

We quantified the total stream length of each stream order and the area of the four wetland types protected in each of the IUCN categories II, III, and VI. We were particularly interested in determining the degree to which adjacent sub-catchments, wetland types and fish species were represented under IUCN category II, as this is the highest level of protection afforded to any terrestrial area in the Wet Tropics. We determined the percent of total adjacent sub-catchment and wetland area fully within an IUCN category II protected area (i.e., the entire sub-catchment and wetland area were protected). We then determined the percent of the distribution of the 45 fish species in the Wet Tropics that is represented in IUCN category II protected areas. As a benchmark, we evaluated how many of the Strahler stream orders, each wetland type and each fish species had at least 20% of their total length, area or distribution within the Wet Tropics represented within a protected area. While useful for comparisons, it is important to note that (1) the use of a uniform percentage target gives equal importance to all ecosystems and species and (2) equal importance is not always used in conservation planning.

### Quantifying human-induced disturbances

For each stream order we determined the percent of total adjacent sub-catchment area occupied by the seven land uses. To quantify the number of human-induced disturbances influencing fresh waters and fish species, we determined: 1) the percent of total adjacent sub-catchments (n = 7210) where olive hymenachne, pond apple and salvinia infestations are present; 2) the percent of total adjacent sub-catchments with 0, 1, 2, 3, 4, 5 or 6 of the human-induced disturbances present (urban/residential areas, grazing, intensive agriculture or horticulture, presence of olive hymenachne, pond apple or salvinia); and 3) the percent of adjacent sub-catchments, where the four wetland types occur, with 0, 1, 2, 3, 4, 5 or 6 of the prominent disturbances. Finally, we established the total stream reach length, adjacent sub-catchment area and number of stream reaches upstream of any protected area, irrespective of the protection level, with each level (0–6) of the human-induced disturbances present.

## Supporting Information

Figure S1 **Strahler stream order and fish richness.** Distribution of a) Strahler stream orders 1–6 and b) fish species richness by stream reach (n = 7210), based on modeled distributions for 45 fish species.
(TIF)

Table S1 **Validation of modeled fish distributions.** The Area Under the Receiver Operator Characteristic (ROC) curve (AUC) for distribution models established for 45 freshwater fish species. *  =  species endemic to the Wet Tropics.
(DOCX)
