# Supplementary figures and images for: Fresh Waters and Fish Diversity: Distribution, Protection and Disturbance in Tropical Australia
Source: PLoS One. 2011 Oct 6;6(10):e25846. doi: 10.1371/journal.pone.0025846 (PMC3188569; doi:10.1371/journal.pone.0025846)

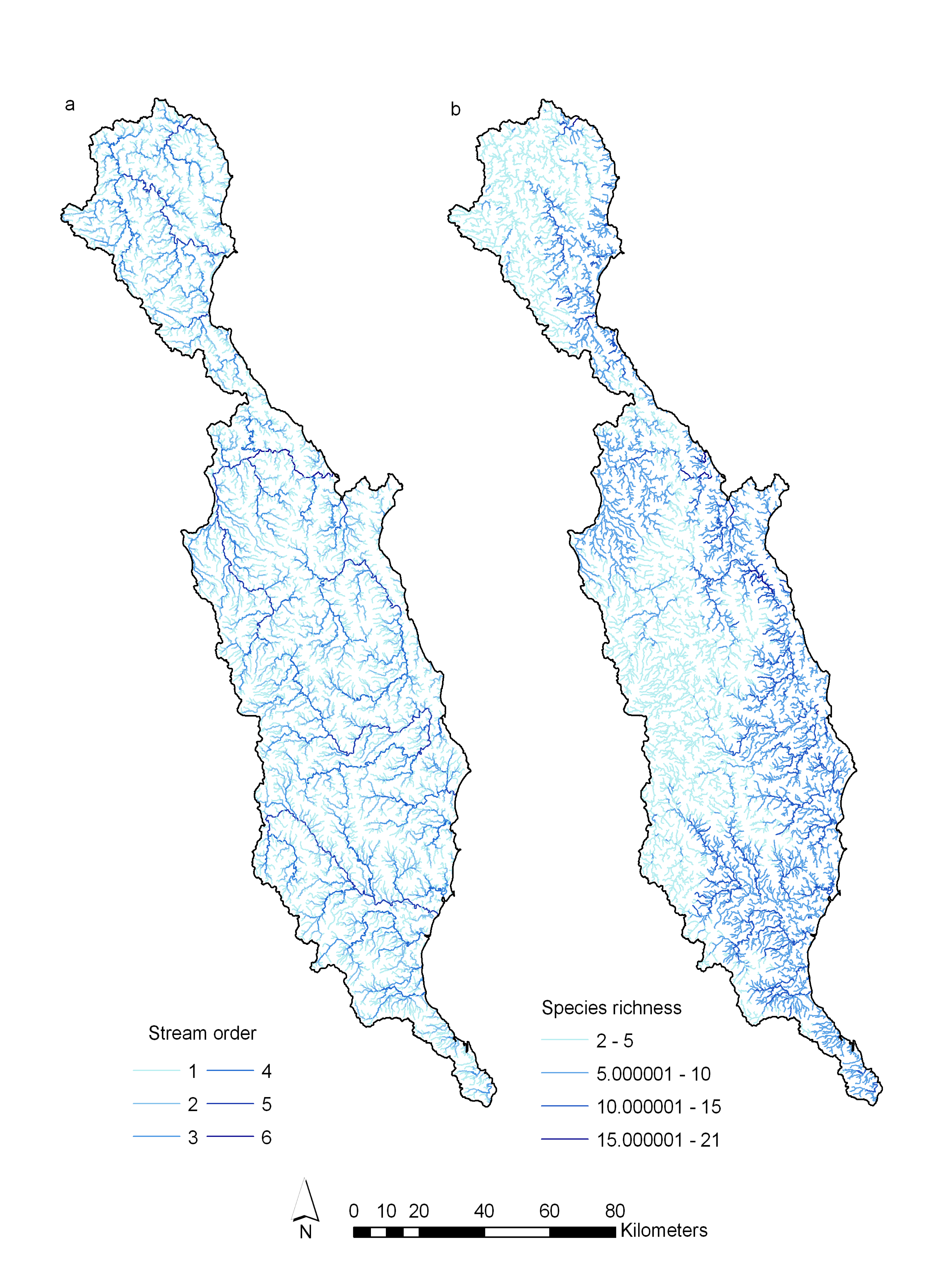

Supplement: Figure S1 — Strahler stream order and fish richness. Distribution of a) Strahler stream orders 1–6 and b) fish species richness by stream reach (n = 7210), based on modeled distributions for 45 fish species. (TIF) [file pone.0025846.s001.tif]
