# Supplementary material for: Fresh Waters and Fish Diversity: Distribution, Protection and Disturbance in Tropical Australia
Source: PLoS One. 2011 Oct 6;6(10):e25846. doi: 10.1371/journal.pone.0025846 (PMC3188569; doi:10.1371/journal.pone.0025846)
Supplement: Supplementary file 2 [file pone.0025846.s002.docx]

Neosilurus ater	0.83
Neosilurus hyrtlii	0.96
Notesthes robusta	0.79
Ophisternon gutturale	0.89
Oxyeleotris aruensis	0.79
Oxyeleotris lineolatus	0.85
Pseudomugil gertrudae	0.86
Porochilus rendahli	0.89
Pseudomugil signifer	0.89
Redigobius bikolanus	0.77
*Schismatogobius sp.**	0.82
Tandanus tandanus	0.73
Toxotes chatareus	0.91
